# Proline *cis-trans* isomerization is influenced by local lysine acetylation-deacetylation

**DOI:** 10.15698/mic2014.11.176

**Published:** 2014-01-23

**Authors:** Françoise S. Howe, Jane Mellor

**Affiliations:** 1 Department of Biochemistry, University of Oxford, South Parks Road, Oxford, OX1 3QU, UK.

**Keywords:** lysine acetylation, proline cis-trans isomerization, histone H3, intrinsically disordered regions, post-translational modifications, protein structure, Saccharomyces cerevisiae

## Abstract

Acetylation of lysine residues has several characterised functions in chromatin. These include neutralization of the lysine’s positive charge to directly influence histone tail-DNA/internucleosomal interactions or indirect effects via bromodomain-containing effector proteins. Recently, we described a novel function of lysine acetylation to influence proline isomerization and thus local protein conformation. We found that acetylation of lysine 14 in the histone H3 N-terminal tail (H3K14ac), an intrinsically disordered domain, increased the proportion of neighbouring proline 16 (H3P16) in the *trans* conformation. This conformation of the tail was associated with reduced tri-methylation on histone H3 lysine 4 (H3K4me3) due to both decreased methylation by the Set1 methyltransferase (with the me3-specific subunit Spp1) and increased demethylation by the demethylase Jhd2. Interestingly, H3K4me3 on individual genes was differentially affected by substitution of H3K14 or H3P16, with ribosomal protein genes losing the least H3K4me3 and environmental stress-induced genes losing the most.

 The tails of histone proteins have traditionally been thought of as unstructured due to their absence from the X-ray crystal structure. However, from molecular dynamics simulations and circular dichroism, it is becoming clearer that structures such as helices can form dynamically within these regions. Proline, with its unique ability to form *cis* or *trans* peptide bonds with the neighbouring residue, provides a potential hinge point to these structures (Fig. 1A).

**Figure 1:**
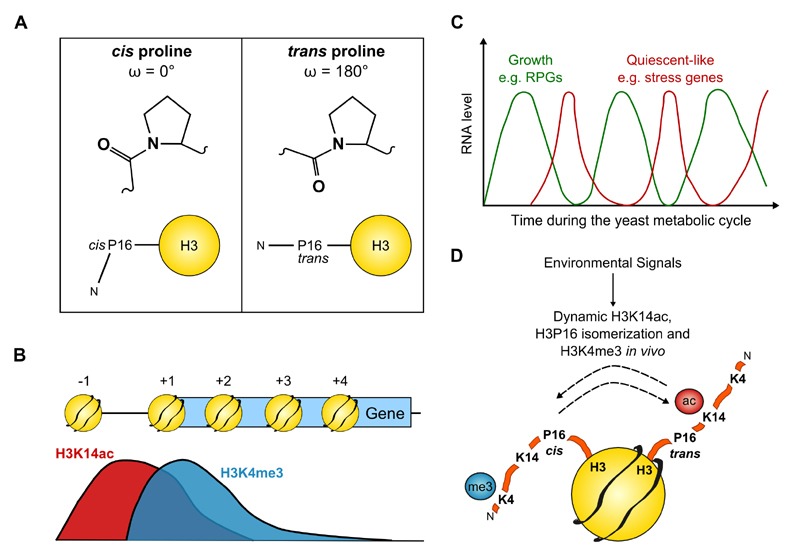
FIGURE 1: **(A)** Structures of *cis *and *trans* proline and a schematic of the histone H3 N-terminal tail with proline 16 in the two possible conformations. **(B)** Distribution of H3K14ac and H3K4me3 across a typical gene in yeast. **(C)** The changes in RNA level of oxidative (green) and reductive (red) genes during the yeast metabolic cycle. **(D)** Environmental signals lead to dynamic H3K14ac, H3P16 isomerization and altered H3K4me3 *in vivo*.

We raised and characterised antibodies against the *cis* and *trans *forms of proline 16 in the histone H3 tail, making use of the fact that hydroxylating the proline ring can fix the peptidyl-prolyl bond in either the *cis* or *trans* conformations. We used these antibodies in a chromatin immunoprecipitation (ChIP) experiment to show that H3P16*cis* is 5’-localised whereas H3P16*trans* increases towards the 3’ end of* FMP27*, a long, transcribed gene in yeast. Since the localization of H3P16*cis* was similar to that of H3K4me3, we asked whether H3P16 is required for H3K4me3. Substitution of H3P16 with valine, to mimic the *trans* state, reduced levels of H3K4me3 both globally and on individual genes, indicating that H3P16*cis* or the ability to isomerize H3P16 was required for optimal H3K4me3. We knew from our work and others that neighbouring H3K14 is also required for optimal H3K4me3, but with three different substitutions (alanine, arginine or glutamine) resulting in altered residual H3K4me3: a K14R strain had near-wildtype (WT) levels of H3K4me3 (79%) whereas the K14A and K14Q strains had 26% and 43% of WT levels respectively (Fig. 1B).

ChIP-sequencing revealed that H3K4me3 was affected similarly genome-wide by substitution of H3K14 with alanine or H3P16 with valine but that H3K4me3 on individual genes had differing levels of H3K14/H3P16 sensitivity. Indeed, on a small proportion of genes, H3K4me3 was present at near-WT levels, indicating that neither H3K14 nor H3P16 are essential for H3K4me3. Interestingly, similar levels of H3K4me3 were retained on the same genes in the absence of *SPP1*. The H3K14/H3P16/*SPP1*-independent genes were enriched in ribosomal protein genes and genes expressed during the oxidative phase of the yeast metabolic cycle whereas the genes with H3K4me3 most dependent on H3K14/H3P16/*SPP1* are induced during the environmental stress response and expressed during the reductive phase of the metabolic cycle. It is clear that the genes at opposite ends of the H3K14/H3P16/*SPP1*-dependency spectrum function in very different pathways and are unlikely to be co-expressed in the same cell at the same time (Fig. 1C).

Given the similarities in the effects of H3K14 or H3P16 substitution on H3K4me3, we wanted to know whether H3K14 and H3P16 are functionally linked, potentially by neighbouring lysine 14 acetylation influencing proline 16 isomerization. Firstly we exploited the fact that the bromodomain from the SAGA component Spt7 preferentially binds to an H3 peptide containing K18ac when P16 is substituted with valine (and so fixed in the *trans* conformation) compared to the control K18ac peptide (which contains a mixture of P16*cis*/*trans*). Since the Spt7 bromodomain did not bind to an H3 peptide containing K14ac, we could use the binding of this domain to K18ac as readout for the effect of H3K14 acetylation on the isomerization of H3P16. We found that substitution of K14 for arginine, to mimic the unmodified lysine state, abolished the binding of the Spt7 bromodomain to K18ac, but that this binding could be rescued by additional substitution of P16 with valine. This informed us that the reason binding was lost in the K14R-K18ac peptide was due to the adoption of an unfavourable P16 conformation for Spt7 bromodomain binding: the *cis* conformation. Conversely, K14ac increased bromodomain binding and therefore promotes P16*trans*. This was confirmed by chymotrypsin-coupled isomerization assays in which chymotrypsin is only able to cleave and release a coloured product when a neighbouring proline is in the *trans* conformation.

This information allowed us to propose a model (Fig. 1D) in which H3K14ac (or substitution of H3K14 with A or Q) promotes H3P16*trans*, which in turn reduces H3K4me3. Conversely, unmodified H3K14 (or substitution with R) increases the proportion of H3P16*cis* to maintain H3K4me3. Substitutions at H3K14 and H3P16 affected the levels of H3K4me3 both by causing decreased H3K4 tri-methylation and increased demethylation. It is easy to imagine how isomerization of a proline residue in the middle of the H3 tail may alter the proximity of the target residue and the enzyme bound to its docking site. Indeed, extending this concept, lysine acetylation in the vicinity of a proline may act as a more general switch in other proteins with intrinsically disordered regions to seed alternative conformations and mediate interactions with distinct partner proteins. An example is Tau, a microtubule (MT) associated protein that promotes neuronal survival through regulated dynamic binding and stabilization of MTs. Inappropriate acetylation or phosphorylation of Tau contributes to loss of MT binding and the formation of insoluble Tau aggregates in Alzheimer's disease (AD) and related tauopathies. Interestingly, the conformation of a local peptidyl-prolyl bond has also been identified in the regulation of Tau function, specifically proline 232-directed control of phosphorylation at threonine 231. *cis*-phosphorylated Tau cannot promote microtubule assembly, is more resistant to dephosphorylation and degradation, is prone to aggregation and is associated with cognitive impairment in AD. Given the link we have discovered between lysine acetylation and proline conformation in histone H3, it is notable that the proline-rich region of Tau is also subject to lysine acetylation, raising the interesting idea that Tau peptidyl-prolyl bond conformations and function might also be influenced by local lysine acetylation/deacetylation.

Many transcription factors have proline-rich regions with acetylated lysines. One of the best studied is the peroxisome proliferator-activated receptor γ coactivator 1α (PGC-1α), whose acetylation status is regulated by the lysine acetyltransferase (KAT) GCN5 and the lysine deacetylase (HDAC) SIRT1, to coordinate mitochondrial energy homeostasis and energy levels. Four of the acetylation sites clustered in the centre of the protein are in proximity to proline residues, again raising the possibility that lysine acetylation/deacetylation might alter proline isomerization and thus interactions with other activators, repressors or co-regulators of transcription. Levels of lysine acetylation are reflected in the metabolic state of a cell, particularly the co-factors, acetyl-CoA and NAD+ for the KATs and HDACs of the sirtuin class, respectively. Thus, diet and the intrinsic metabolic cycles that regulate the production of these co-factors are likely to impinge on the dynamic yet balanced actions of the KATs and HDACs. While the links between proline isomerization and local phosphorylation/dephosphorylation are clear, this work establishes lysine acetylation/deacetylation as a mechanism for switching structures in proteins by influencing proline conformation. As phosphorylation also influences local lysine acetylation, the dynamic regulation of protein conformations and interactions may be subject to wide-ranging metabolic, developmental and stress-related signals.

